# Morphological Evidence for Functional Crosstalk Between Multiple GnRH Systems in the Male Tilapia, *Oreochromis niloticus*

**DOI:** 10.3389/fendo.2020.00586

**Published:** 2020-09-02

**Authors:** Satoshi Ogawa, Ishwar Parhar

**Affiliations:** Brain Research Institute, Jeffrey Cheah School of Medicine and Health Sciences, Monash University Malaysia, Bandar Sunway, Malaysia

**Keywords:** GnRH, GAP, GnRHR, cichlid, reproduction

## Abstract

Gonadotropin-releasing hormone (GnRH) is a reproductive neuropeptide, which controls vertebrate reproduction. In most vertebrates, there are more than two GnRH orthologs in the brain. In cichlid fish, the Nile tilapia (*Oreochromis niloticus*), GnRH1 is the primary hypophysiotropic hormone, while GnRH2 and GnRH3 are non-hypophysiotropic but neuromodulatory in function. Hypophysiotropic GnRH neurons are thought to inter-communicate, while it remains unknown if hypophysiotropic and non-hypophysiotropic GnRH systems communicate with each other. In the present study, we examined interrelationship between three GnRH types using specific antibodies raised against their respective GnRH associated peptide (GAP) sequence. Double-immunofluorescence labeling coupled with confocal microscopy revealed that in sexually mature males, GnRH-GAP1-immunoreactive (-ir) processes are in proximities of GnRH-GAP3-ir cell somata in the terminal nerve, while GnRH-GAP1-ir cell somata were also accompanied by GnRH-GAP3-ir processes in the preoptic area. However, such interaction was not seen in immature males. Further, there was no interaction between GnRH-GAP2 and GnRH-GAP1 or GnRH-GAP3 neurons. Single cell gene expression analysis revealed co-expression of multiple GnRH receptor genes (*gnrhr1* and *gnrhr2*) in three GnRH-GAP cell types. In mature males, high levels of *gnrhr2* mRNA were expressed in GnRH-GAP1-ir cells. In immature males, *gnrhr1* and *gnrhr2* mRNAs are highly expressed in GnRH-GAP3-ir cells. These results suggest heterologous interactions between the three GnRH-GAP cell types and their potential functional interaction during different reproductive stages.

## Introduction

Gonadotropin-releasing hormone (GnRH) is a neuropeptide that primarily plays an essential role in the control of reproduction in vertebrates (1–5). GnRH stimulates the release of gonadotropins: follicle-stimulating hormone (FSH for gamete growth) and luteinizing hormone (LH for gamete maturation and release) from the adenohypophysis or anterior pituitary (6). Most vertebrate species possess more than two different GnRH types and their receptor (GnRH receptor, GnRHR) orthologs (7, 8). In mammals, there are two GnRH types, GnRH1 (mammalian GnRH) and GnRH2 (chicken-GnRH-II). GnRH1 is primarily hypophysiotropic in function and is the species-specific form (7, 9). On the other hand, GnRH2 is structurally most conserved in vertebrates and is expressed in the midbrain region. In addition to these two GnRH types, the third type of GnRH (GnRH3 = salmon GnRH) is present in the forebrain of teleosts. Similarly, most vertebrates possess more than two GnRHR types. For example, mammalian species have two forms of GnRHR, while amphibian and reptilian species have three forms of GnRHR (10). In fish, GnRHR orthologs have been classified into three major lineages of GnRHR types that are further subdivide into five classes: non-mammalian type I (GnRHRn1 and GnRHRn1b), non-mammalian type II (GnRHRn2), and non-mammalian type III (GnRHRn3 and GnRHRn3b) (11).

Differential distribution of GnRH and GnRHR types in several brain regions indicate their involvement in a variety of functions that are directly or indirectly associated with reproduction. In mammals, GnRH1 neurons located within the hypothalamic regions send neuronal processes mainly to the median eminence to control gonadotropin release. However, GnRH1 neuronal processes as well as GnRHR are also found in extrahypothalamic regions such as the hippocampus, amygdala, striatum, septum, thalamic nuclei, midbrain tectum and periaqueductal gray region, and brainstem (12, 13). GnRH1-GnRHR pathway has been implicated in functions such as cognition and mood, which are associated with reproductive stages such as puberty and menopause in human, mice and sheep (14–16).

Although GnRH2 can pharmacologically stimulate LH release, GnRH2 has been speculated to have originated in the immune system and it has recently evolved as a neuromodulator (17). In several vertebrates, GnRH2 stimulates sexual behavior (18–20) and in the musk shrew, GnRH2 suppresses feeding behavior (21). Similarly, in the zebrafish, central administration of GnRH2 peptides reduce food intake (22), and GnRH2 gene knockout zebrafish exhibit increased feeding and growth (23). The fish specific form, GnRH3 is mainly localized in the terminal nerve region in the forebrain and is involved in a variety of roles depending on the fish species. In some fish species, such as the goldfish and zebrafish, GnRH3 is expressed in the terminal nerve and in the preoptic area, indicating its hypophysiotropic and neuromodulatory functions (24, 25). GnRH3 in the terminal nerve regions regulates social behaviors such as aggression, sexual behavior, and partner preference (26–28). Although the hypophysiotropic GnRH form is primarily essential for the LH secretion, but other functions such as social behaviors, food intake, and mood that are modulated by non-hypothalamic GnRH forms are also closely associated with reproductive conditions.

It is clear that socio-sexual behaviors and mental status that are known to be influenced by GnRH1, are closely associated with the reproductive status (14, 29). However, it remains unknown how GnRH neurons control non-hypophysiotropic functions in the extrahypothalamic regions. Do the same GnRH neurons send neuronal projections to the adenohypophysis and to other brain regions? How do GnRH neurons co-modulate reproduction (LH surge) and its associated functions? Is there a mechanism/pathway to connect these functions? Hypothalamic GnRH1 neurons coordinate amongst themselves through synaptic interactions and gap junctions (30), which suggest an autocrine/paracrine regulation (31). On the other hand, other functions that are affected by non-hypothalamic GnRH systems, such as metabolic status or sexual behaviors are also closely associated with hypophysiotropic GnRH function. However, it is unknown if hypophysiotropic GnRH form can communicate with non-hypothalamic GnRH forms.

To elucidate the possible functional interaction between different GnRH systems, we used a cichlid fish, Nile tilapia (*Oreochromis niloticus*), which possesses three GnRH and GnRHR types (32). In the brain of the Nile tilapia and other perciforms, GnRH1 is localized in the preoptic-hypothalamic regions, GnRH2 is in the midbrain tegmentum and GnRH3 is mainly in the terminal nerve, but there is some overlap in the distribution between GnRH1 and GnRH3 cell somata/processes in the forebrain and the terminal nerve-preoptic region (33–36). In tilapia, three GnRHR (GnRHR1, GnRHR2, and GnRHR3) types have been identified, among which, GnRHR1 and GnRHR3 are classified as GnRHRn1, while GnRHR2 is classified as GnRHRn3 based on genome synteny analysis and sequence similarity (11, 37). Interrelationship between the three GnRH cell types were examined using specific antisera for GnRH associated peptide (GAP) derived from GnRH precursor, which is coexpressed with GnRH (33, 35, 36) (and thus hereafter referred to as “GnRH-GAP”). Further, to investigate the expression of multiple GnRHR types in three GnRH-GAP cell types (GnRH-GAP1, GnRH-GAP2, and GnRH-GAP3) and their potential involvement under different reproductive stages, expression of three GnRHR types (*gnrhr1, gnrhr2*, and *gnrhr3*) mRNA levels were examined in microdissected three GnRH-GAP cell types in immature and mature males of tilapia.

## Materials and Methods

### Animals

Nile tilapia (*Oreochromis niloticus*) were maintained in freshwater aquaria at 27 ± 1°C with a controlled natural photo-regimen (14/10 h, light/dark). Juvenile fish (2–3 weeks after fertilization) were obtained from the fish facility at Tokyo University of Marine Science and Technology (Tokyo, Japan) and were reared until the experimental sizes [sexually immature: standard length (SL), 4.4 ± 0.07 cm; body weight (BW), 3.2 ± 0.2 g; gonado-somatic index (GSI), 0.032 ± 0.018%, and mature: SL, 14.6 ± 0.2 cm; BW, 92.6 ± 4.5 g, GSI, 1.6 ± 0.4%] in the fish facility at Nippon Medical School (Tokyo, Japan). Sexually mature males were placed in a 60 L tank with two polyvinyl chloride (PVC) pipes (10 cm in diameter and 15 cm in length) as shelters for subordinate males to reduce social stress, which also prevented territory holding and reduced territorial behavior. The fish were fed commercial fish diet once a day. Fish were anesthetized by immersion in a 0.01% solution of 3-aminobenzonic acid ethyl ester (MS222; Sigma, St. Louis, MO, USA) before they were killed by decapitation. The fish were maintained and used in accordance with the Guidelines of the Animal Ethics Committees of Nippon Medical School.

### Tilapia GAP Antisera Production and Specificity Characterization

Possible association of neuronal processes (axon/dendrites) and cell somata between the three GnRH-GAP types in the brain were examined using specific antibodies against the tilapia GAP types (Antibody codes: #ISP105 for GAP1, #ISP205 for GAP2, and #ISP305 for GAP3) (38–40). The antisera were generated in rabbits against commercially synthesized tilapia GAP peptides (GAP1: amino acids 63-94; GAP2, amino acids 55-94; GAP3, amino acids 46-85; GenBank Accession nos., AB101665, AB101666, and AB101667, respectively) (T.K. Craft Co., Ltd, Japan). After final blood collection, the blood was centrifuged and the affinity-purified serum was stored at – 80°C.

Specificity of the GAP antisera were evaluated by the Western blot analysis (see below). In addition, the GAP antisera immunoreactivities were also verified by GnRH-immunoreactivities and gene expression patterns of three GnRH types (*gnrh1, gnrh2*, and *gnrh3*) in the brain (41, 42). For controls for immunohistochemistry, brain sections of adult tilapia were incubated with the GAP antisera that have been preabsorbed with respective antigen (see below).

#### Western Blot Analysis

Western blot analysis was performed according to Pandolfi et al. (36) with a slight modification. Sexually mature male tilapias (*n* = 3) were anesthetized by immersing into 0.01% tricaine methanesulfonate (MS222; Sigma, St. Louis, MO, USA) solution. The brains and pituitaries were separately dissected out and homogenized in 1 ml and 100 μl of 50 mM Tris-HCl buffer (pH 7.4) containing 10 μl per ml protease inhibitor mix (GE Healthcare Life Sciences, Piscataway, NJ), respectively. The soluble fractions were obtained by centrifugation at 6,000 G for 30 min at 4°C. The protein concentration was measured by the Lowry assay (Bio-Rad, Hercules, CA). An aliquot of tissue sample was diluted with an equal volume of Laemmli's sample buffer (Bio-Rad). Supernatants were separated by 15% polyacrylamide gel with 20 μg protein per lane along with the marker (ECL DualVue Western Blotting Markers; GE Healthcare Life Sciences). After electrophoresis, the proteins were transferred onto a polyvinylidene difluoride membrane (Hybond-P, GE Healthcare Life Sciences) in Tris/glycine buffer (25 mM Tris, 192 mM glycine), and 20% methanol for 30 min at 40 mA. The membranes were washed in Tris-buffered saline (pH 7.4) with 1% Tween-20 (TBST) and blocked with TBST containing 0.5% non-fat dry milk for 2 h at room temperature, and washed with TBST three times at 5 min intervals and then incubated with GAP antisera at dilution of 1:1000 (#ISP105 and #ISP205) and 1:3000 (#ISP306) for overnight at 4°C. The membrane was washed in TBST and then incubated with horseradish peroxidase (HRP)-conjugated donkey anti-rabbit IgG (GE Healthcare Cat# NA934-100Ul) at room temperature for 1 h. ECL Plus Western Blotting Detection Reagents (GE Healthcare) was used to detect antibody binding and chemiluminofluorescence signals were visualized with the Light-Capture System attached to a cool CCD camera (ATTO, Tokyo, Japan).

#### Immunohistochemistry

The dissected brains (*n* = 3) were fixed in Bouin's solution, and dehydrated through graded series of ethanols, cleared in n-butanol and embedded in Paraplast Plus (Oxford Labware, St Louis, MO, USA). Serial brain sections were cut in the sagittal plane (15 μm) and processed for immunohistochemistry. The rabbit anti-tilapia GAP antiserum (dilution of 1:1000–3000) were prepared in PBS containing 2% normal goat serum, and 0.5% Triton X-100, and the control anti-GAP antisera were preabsorbed with 10 μg/ml of respective antigen at the working dilution for 24 h. Alternate brain sections were incubated with primary GAP antiserum and pre-absorbed primary antiserum (*n* = 1 for each antiserum) at 4°C for 24 h in a closed moist chamber. Diluted biotinylated anti-rabbit IgG and avidin-biotinylated horse radish peroxidase (HRP) complex (Vectastain ABC Elite kit, Vector Laboratories Cat# PK-6101) were then applied to the sections for 30 and 45 min, respectively. Development was achieved through application of 0.05% 3,3-diaminobenzidine tetrahydrochloride (Sigma) with 0.03% H_2_O_2_ in 0.05 M Tris-HCl (pH 7.5). After dehydration, stained sections were cleared in xylene, coverslipped, and sealed with DPX mounting medium (Fisher Scientific, Loughborough, UK). Sections were scanned and images were captured with a Carl Zeiss MIRAX slide scanning system (Zeiss GmbH, Göttingen, Germany) using the Mirax Viewer Image Software (3DTech, Budapest, Hungary) at a 230 nm resolution with a × 20 objective.

### Double-Immunofluorescence of GnRH-GAP Types

Brain of sexually immature (*n* = 3) and mature tilapia (*n* = 3) was dissected and fixed in buffered 4% paraformaldehyde (pH 7.5) for 6–8 h, cryoprotected in 20% sucrose in 0.1 M phosphate buffer and embedded in Tissue Tek OCT compound (Sakura Finetechnical, Tokyo, Japan). Sagittal sections (30 μm) were cut on a cryostat and thaw-mounted onto APS-coated slide glass. Sections were incubated for 24 h at 4°C with GAP antisera and incubated for 2 h with Alexa Fluor 488- or 594-labeled anti-rabbit IgG (Invitrogen, dilution of 1:400). Since double-label immunohistochemistry using the antisera generated in the same animal species can potentially result in cross-reactivity, we used F(ab)^2^ fragments anti-rabbit IgG to block the free-binding sites of secondary antibody (43). After several washes in PBS, the sections were incubated for 2 h in 2% normal rabbit serum at room temperature, and incubated for 2 h with F(ab')2 fragment anti-rabbit IgG (Jackson Immunoresearch Laboratories, Inc., PA, USA; dilution of 1:100). Sections were then incubated for 24 h at 4°C with different types of GAP antisera to be paired with different GnRH-GAP types (GAP1-GAP2, GAP2-GAP3, and GAP3-GAP1), and incubated for 2 h with Alexa Fluore 488- or 594-labeled anti-rabbit IgG (Invitrogen). Coverslips were applied with Vectashield medium (Vector Laboratories, Burlingame, CA) and dual-label immunofluorescent images were obtained using a fluorescent microscope (Nikon, Eclips 90i, Nikon) attached with a cooled CCD camera.

Detailed structures were further analyzed under a laser confocal microscope (C1si, Nikon Instruments, Tokyo, Japan) using laser wavelength of 488 and 543 nm. The images were captured and superimposed automatically by an imaging software (NIS Elements, Nikon). The red channel was converted to magenta, and brightness and contrast adjustments were made in Adobe Photoshop CC (Adobe, San Jose, CA, USA).

### Single Cell Dissection of GnRH-GAP Cells and RNA Extraction

Single cell harvesting of GnRH-GAP-immunoreactive cells and total RNA extraction was performed as described previously with slight modifications (38, 39, 42, 44). Briefly, the brain sections were immunoreacted with GAP antiserum for overnight followed by incubation with secondary antibodies (AlexaFluor 488- or 594-labeled anti-rabbit IgG) as described above in RNase-free buffer. GnRH-GAP-immunolabeled cells were microdissected by heat-pulled borosilicate glass micro capillary pipette (1.5 mm outer diameter, Harvard Apparatus Ltd., Edenbridge, Kent; micropipette puller; Type PE-2, Narishige, Tokyo, Japan) attached to a micromanipulator (Narishige). Then, single GnRH-GAP cells were harvested by the micropipette and collected into a sterile 1.5 ml reaction tube containing 50 μl of the lysis buffer. The harvested single GAP cell was digested with 1 μg of proteinase K (Gentra Systems, Minneapolis, MN) for 1 h at 53°C. The cell lysate was incubated for 1 h at 37°C with 1U RNase-free DNase I (Promega, Madison, WI) to eliminate genomic DNA. Total RNA was extracted from the cell lysate using ISOGEN (Nippon Gene, Tokyo, Japan) and reverse transcribed to cDNA in a reaction mixture (20 μl) with 100 pmol of random primers (Takara, Tokyo, Japan) and 40U SuperScript III Reverse Transcriptase (Invitrogen, Carlsbad, CA).

### Quantitative Analysis of GnRH and GnRHR Types mRNAs in GnRH-GAP Cells

To quantify mRNA levels of *gnrhr* types in GnRH-GAP cells, single GnRH-GAP cell's cDNA samples were subjected to real-time PCR for *gnrh1, gnrh2, gnrh3, gnrhr1, gnrhr2*, and *gnrhr3* with an ABI PRISM 7700 Sequence Detection System (PE Applied Biosystems). The PCR mixture (10 μl) contained 1 × TaqMan Universal PCR Master Mix (Applied Biosystems), 300 nM of forward and reverse primers (Supplementary Table 1), 200 nM of hybridization probe (Supplementary Table 1), and one 20th of a single cell's cDNA or absolute standard cDNA. All primers and probes were designed to span intron-exon junction, and thus, no amplification of genomic sequence was seen in the cDNA samples. The PCR conditions were 95°C for 10 min, 60 cycles at 95°C for 15 s and 60°C for 1 min. The absolute amounts of transcripts were determined by establishing a linear amplification curve from serial dilutions (10^1^ to 10^6^ fg) of a plasmid DNA containing a *gnrh* or a *gnrhr* partial cDNA sequence. For each animal, the average mRNA levels of *gnrh* types and *gnrhr* per cell were determined and these values were combined to give experimental group means. For GnRH-GAP cells, expression of glial fibrillary acidic protein (*gfap*) gene (Supplementary Table 1) was also examined, and only those cells that tested negative for *gfap* but positive for respective *gnrh* genes were included for data analyses. All values are presented as the means (±SEM). Correlation of *gnrh* and *gnrhr* types mRNA levels were examined by Pearson R, while the mean difference of *gnrh* and *gnrhr* types mRNA levels between mature and immature were analyzed by unpaired Student's *t*-test or non-parametric ANOVA followed by *post-hoc* Dunn's multiple comparison test. The Tukey's criterion applied to each group of data sets, allowing detection of outliers (45). *P* < 0.05 was considered statistically significant.

## Results

### Antibody Specificity

**Figure 1:**
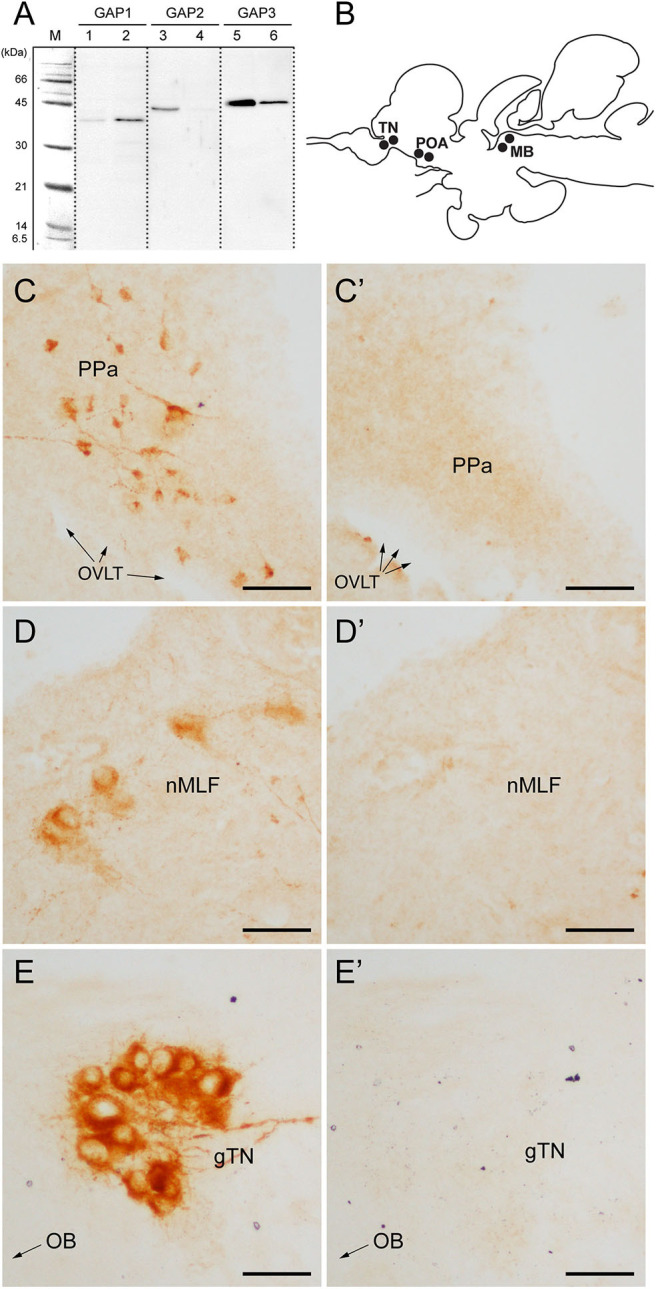
Antibody characterization and immunohistochemical distribution of three GAP-immunoreactivities in the brain of tilapia. **(A)** Western blot analysis of antisera to GAP types (#ISP105 for GAP1, #ISP205 for GAP2, and #ISP306 for GAP3) in the brain (lanes 1, 3 and 5) and pituitary (lanes 2, 4 and 6) homogenate. M, molecular mass marker. **(B)** Schematic diagram of distribution of three GnRH-GAP-immunoreactive cell types in the brain of tilapia: GnRH-GAP1 in the preoptic area (POA), GnRH-GAP2 in the midbrain (MB) and GnRH-GAP3 in the terminal nerve (TN). **(C–E)** Photomicrographs of three GAP-immunoreactivities **(C–E)** and their preaborption with respective GAP antigen **(C'–E')**. PPa, anterior part of the parvocellular preoptic nucleus; OVLT, organum vasculosum laminae terminalis; nMFL, nucleus of the medial longitudinal fascicle; gTN, terminal nerve ganglion; OB, olfactory bulb. Scale bars: 50 μm.

Specificity of antisera to GAP types, brain tissue extracts were analyzed by SDS-PAGE and Western blots. Distinct single bands were detected in the brain and pituitary homogenate reacted with the antisera to GAP types (Figure 1A). A band immunoreactive to GAP2 in the brain was absent in the pituitary. However, detected immunoreactive bands were seen in unexpected sizes ranging from 35 to 45 KDa for all antisera, which are far bigger than the expected sizes of tilapia GAP types (7, 5.7, and 6 kDa for GAP1, GAP2, and GAP3, respectively) or prepro-GnRH types (11.4, 9.6, and 10.1 KDa for tilapia prepro-GnRH1, GnRH2, and GnRH3, respectively).

Using GAP antisera, three distinct GnRH-GAP neural populations were observed (Figure 1B); GnRH-GAP1 cell somata in the ventral telencephalon and anterior part of the parvocellular preoptic nucleus (Figure 1C), GnRH-GAP2 cell somata in the nucleus of the medial longitudinal fasciculus of the midbrain tegmentum (Figure 1D) and GnRH-GAP3 cell somata in the terminal nerve ganglion (also known as the nucleus olfacto-retinalis) (Figure 1E). Parhar et al. (34), Parhar et al. (41) all immunoreactivities in the brain were successfully blocked by the preabsorption with respective antigen peptide (Figures 1C'–E').

### Association Between Neuronal Processes (Axons/Dendrites) and Cell Somata of Three GnRH-GAP Types

Using the GAP antisera, we examined possible association between neuronal processes and cell somata of three GnRH-GAP types in the brain. Since the discrimination between dendrites and axons is technically difficult, we define the labeled dendrites and axons collectively as neuronal processes in the entire manuscript.

**Figure 2:**
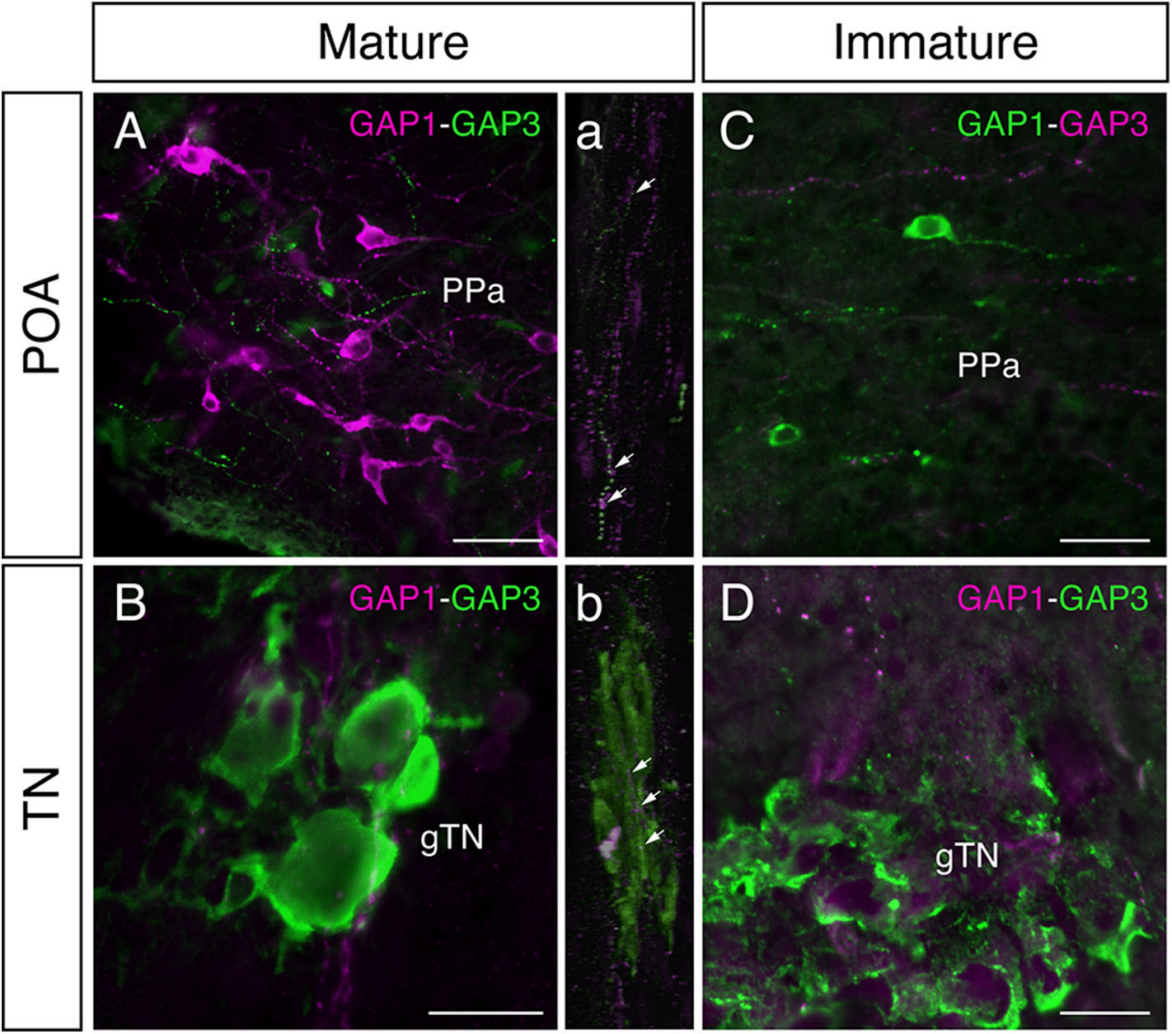
Photomicrographs of double immunofluorescence of GnRH-GAP1 and GnRH-GAP3 in the brain of sexually mature and immature male tilapia. In mature males, GnRH-GAP3-immunoreactive (ir) neuronal processes (green) are closely located to GnRH-GAP1-ir cell somata (magenta) in the anterior part of the parvocellular preoptic nucleus (PPa) of the preoptic area (POA, **A**), while in the terminal nerve ganglion (gTN), GnRH-GAP1-ir neuronal processes (magenta) are also closely located to GnRH-GAP3-ir cell somata (green) the **(B)**. Confocal scanning of these regions further confirmed the proximities of GnRH-GAP1 neuronal processes with GnRH-GAP3 cell somata (a,b). In contrast, neuronal interaction between GnRH-GAP1 and GnRH-GAP3 were not seen in immature males **(C,D)**. Scale bars = 20 μm.

**Figure 3:**
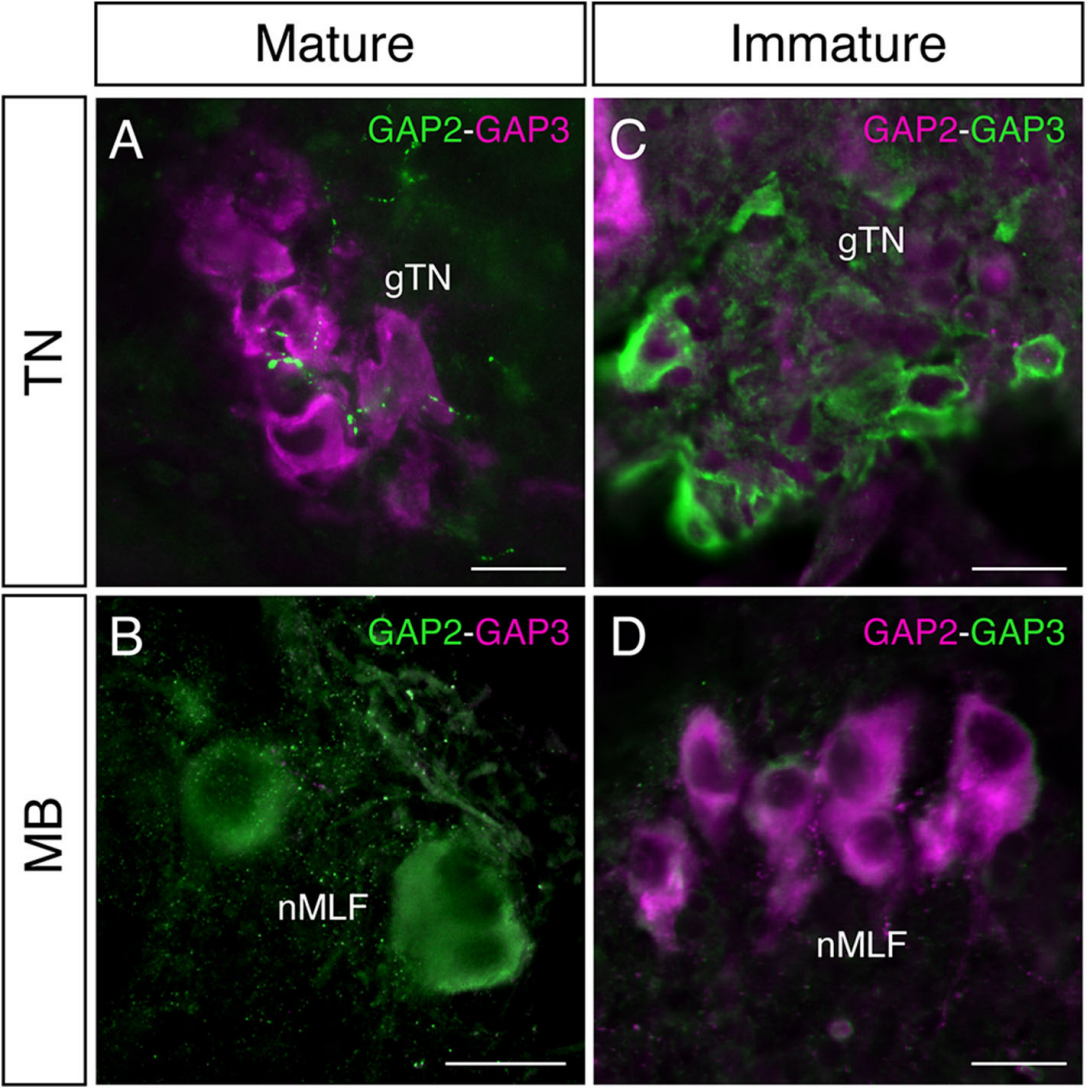
Photomicrographs of double immunofluorescence of GnRH-GAP2 and GnRH-GAP3 in the brain of sexually mature and immature male tilapia. In mature males, GnRH-GAP2-immunoreactive (ir) neuronal processes (green) are closely located to GnRH-GAP3-ir cell somata (magenta) in the terminal nerve ganglion (gTN, **A**), while such interaction between GnRH-GAP3-ir neuronal processes (magenta) and GnRH-GAP2-ir cell soma (green) was not seen in the nucleus of the medial longitudinal fascicle (nMLF) of the midbrain (MB, **B**). No proximities of GnRH-GAP2 (magenta) and GnRH-GAP3 (green) neuronal processes/cell somata were observed in immature males **(C,D)**. Scale bars = 20 μm.

In mature males, GnRH-GAP1-immunoreactive (ir) cell somata are accompanied by closely located GnRH-GAP3-ir neuronal processes in the anterior preoptic area (Figure 2A). Similarly, GnRH-GAP1-ir neuronal processes are in proximities of GnRH-GAP3-ir cell somata in the terminal nerve ganglion (Figure 2B). Confocal imaging further confirmed the proximities of GnRH-GAP1-ir and GnRH-GAP3-ir neuronal processes with cell somata (Figure 2a,b). However, such proximities of GnRH-GAP1 and GnRH-GAP3 neuronal processes with cell somata was not seen in immature fish (Figures 2C,D). There were no proximities of GnRH-GAP1 and GnRH-GAP2 processes/cell somata in mature and immature fish. On the other hand, GnRH-GAP2-ir neuronal processes are in proximities of GnRH-GAP3-ir cell somata in the terminal nerve in mature and immature males (Figures 3A,C), while no GnRH-GAP3-ir neuronal processes were seen in proximities of GnRH-GAP2-ir cell somata in the midbrain (Figures 3B,D).

### Expression of GnRH and GnRHR Types in Single GnRH-GAP Cells

**Figure 4:**
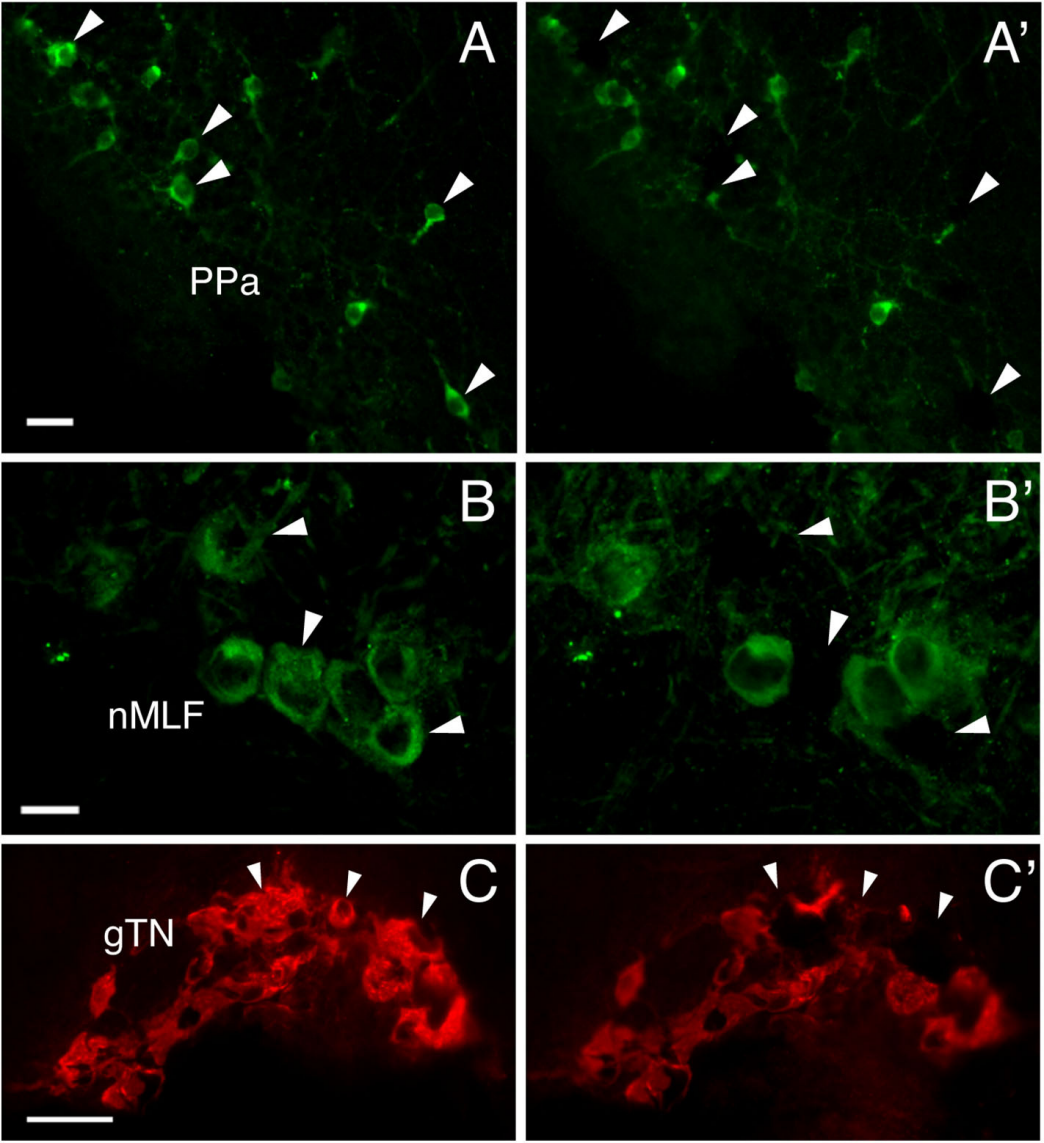
Photomicrographs of GnRH-GAP immunoreactive cell somata before **(A–C)** and after **(A'–C')** microdissection. Arrows indicate harvested cell somata of GnRH-GAP1 **(A,A')** in the anterior part of the parvocellular preoptic nucleus (PPa), GnRH-GAP2 **(B,B')** in the nucleus of the medial longitudinal fascicle (nMLF) and GnRH-GAP3 **(C,C')** in the terminal nerve ganglion (gTN). Scale bars, **(A,B)**, 20 μm; **(C)** 50 μm.

To investigate functional interaction between different GnRH-GAP cell types, expression of GnRHR genes were examined in harvested single-cell GnRH-GAP neurons (Figure 4). All harvested GnRH cell types expressed respective *gnrh* types, *gnrhr1* and *gnrhr2* mRNA transcripts in mature and immature males, while *gnrhr3* mRNA were only detected in few cells. Thus, we only proceeded for quantification of *gnrhr1* and *gnrhr2* mRNAs.

#### GnRH-GAP1

**Figure 5:**
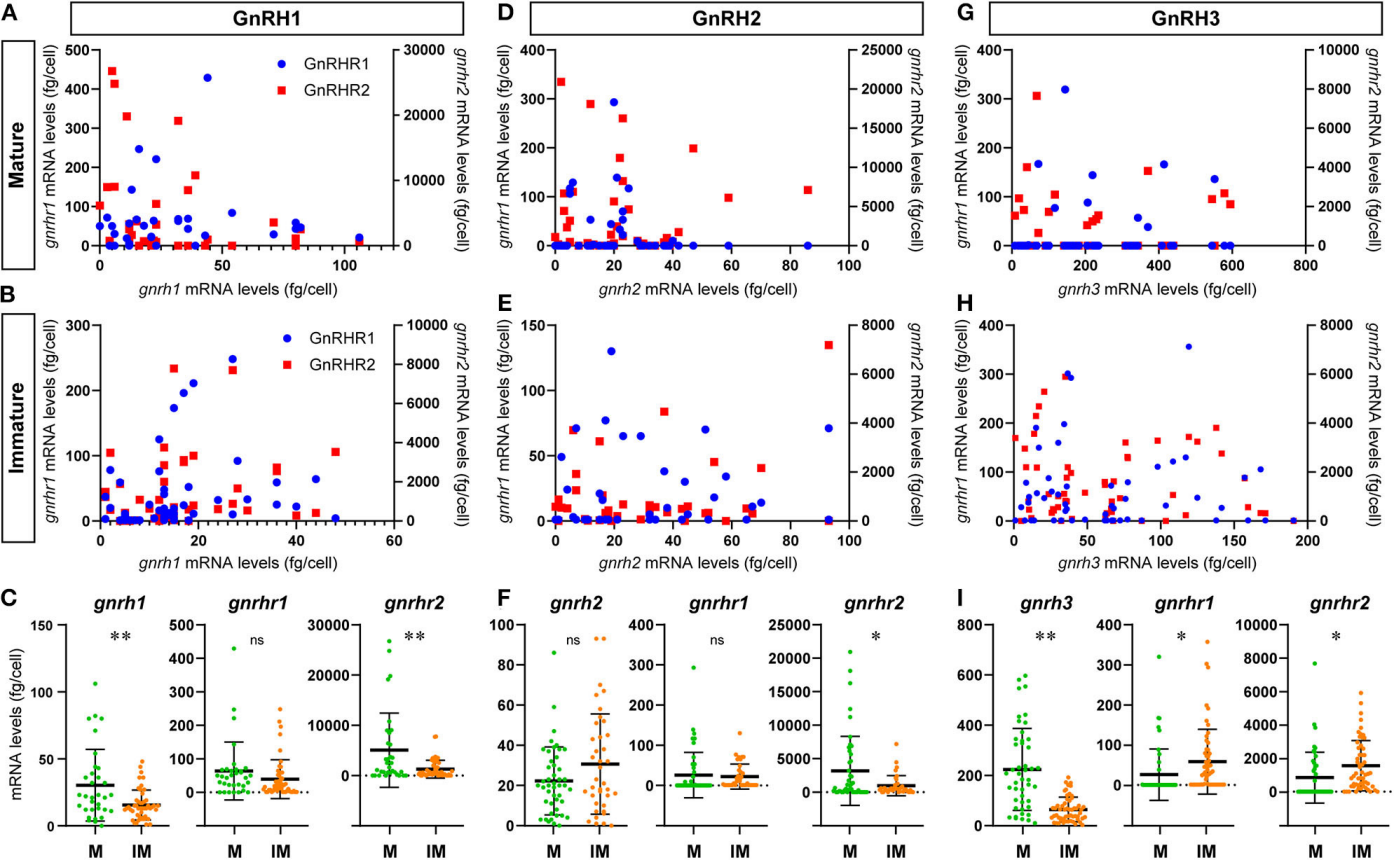
Graphs showing the distribution of GnRH receptor types mRNA transcripts in microdissected single GnRH-GAP neurons in mature and immature males. In the scatter blot diagrams **(A,B,D,E,G,H)**, the x-axis represents the levels of each GnRH mRNAs in GnRH-GAP cell soma, while the 1st (left side) y-axis represents *gnrhr1* mRNA levels and 2nd (right side) y-axis represents *gnrhr2* mRNA levels in each GnRH-GAP cell soma. Red dots represent *gnrhr1* and blue dots represent *gnrhr2* mRNA levels. Column scatter blot diagrams in below **(C,F,I)** show the mRNA levels of GnRH (*gnrh1, gnrh2*, and *gnrh3*) and GnRH receptor types per GnRH-GAP cell soma in mature (M, green) and immature (IM, orange) males. *P < 0.05; **P < 0.001; ns, non-significant.

At the single cell levels, *gnrh1* mRNA levels were significantly (*P* < 0.01) higher in mature (30.4 ± 4.7 copies/cell) than those in immature males (15.5 ± 1.6 copies/cell) (Figure 5C). In mature and immature males, 90–100% of GnRH-GAP1 cells expressing single or multiple GnRHR types (mature, 33/33 cells; immature, 44/49 cells) (Table 1). There was no significant correlation between mRNA levels of *gnrh1* and either *gnrhr1* or *gnrhr2* in immature and mature males (Figures 5A,B). There was no significant difference in *gnrhr1* mRNA levels in GnRH-GAP1 cells between mature and immature males, while *gnrhr2* mRNA levels were significantly (*P* < 0.001) higher in mature males (Figure 5C).

**Table 1:** Percentage of GnRH-GAP1-3 subtypes cells expressing GnRH receptor mRNA transcripts in mature and immature males.

**GnRH-GAP types**	**Maturation stage**	**GnRHR types expression patterns**					
		**GnRHR1 only**	**GnRHR2 only**	**GnRHR3 only**	**Co-expression of any of GnRHR types**	**Co-expression of three GnRHR types**	**No expression**
GnRH-GAP1	Mature	12.1	18.2	0.0	66.7	3.0	0.0
	Immature	0.0	10.2	0.0	81.6	8.2	0.0
GnRH-GAP2	Mature	0.0	34.8	0.0	32.6	2.2	30.4
	Immature	5.3	28.9	0.0	60.5	5.3	0.0
GnRH-GAP3	Mature	10.9	34.8	0.0	8.7	0.0	45.6
	Immature	0.0	29.8	0.0	64.9	1.8	3.5

#### GnRH-GAP2

There was no difference in *gnrh2* mRNA levels between the two reproductive statuses (immature, 30.7 ± 4.1; mature, 22.3 ± 2.5 fg/cell) (Figure 5F). In GnRH-GAP2 cells, 73–95% of cells express single or multiple GnRHR types (mature, 32/46 cells; immature, 36/38 cells) (Table 1). There was no correlation between mRNA levels of *gnrh2* and *gnrhr* types in immature and mature males (Figures 5D,E). There was no difference in *gnrhr1* mRNA levels in GnRH-GAP2 cells between the two reproductive states, while *gnrhr2* mRNA levels were significantly (*P* < 0.05) higher in mature male (3190.0 ± 760.2 fg/ cell) as compared with immature males (961.9 ± 242.2 fg/cell) (Figure 5F).

#### GnRH-GAP3

*gnrh3* mRNA levels were significantly (*P* < 0.0001) higher in mature (221.9 ± 24.1 fg/cell) compared with immature males (66.9 ± 7.7 fg/cell) (Figure 5I). In immature males, high percentage of GnRH-GAP3 cells express *gnrhr* types (55/57 cells), while in mature males, *gnrhr* types expression was only detected in half of cells (24/46 cells) (Table 1). There was no correlation between mRNA levels of *gnrh3* and either *gnrhr1* or *gnrhr2* in immature and mature males (Figures 5G,H). GnRH-GAP3 neurons express significantly (*P* < 0.05) higher mRNA levels of *gnrhr1* and *gnrhr2* in immature (*gnrhr1*, 57.9 ± 10.6 fg/cell; *gnrhr2*, 1525.6 ± 198.5 fg/cell) as compared with mature (*gnrhr1*, 26.7 ± 9.4 copies/cell; *gnrhr2*, 841.3 ± 222.9 copies/cell) males (Figure 5I).

## Discussion

To examine potential functional interaction among GnRH types, we generated three tilapia GAP-specific antisera. Specificity of the three tilapia GAP antisera were characterized by Western blot analysis, which showed a single distinct band of different size for each GAP type, however, the molecular weight for each GAP types was much higher than predicted sizes for GAP types (6–7 kDa) or prepro-GnRH (9–11 KDa). The exact reason for the contradictory results of Western blot assays remains unknown, however, previous studies have also shown high molecular weight of GAP or prepro-GnRH-immunoreactive bands in some fish species. In a cichlid fish, *Cichlasoma dimerus*, Western blot assay for chicken II-GAP antiserum showed a band of 34 kDa in the brain extract (36). Similarly, in clownfish *(Amphiprion melanopus)*, a band of 52 KDa was detected by LRH13, a monoclonal antibody to mammalian GnRH1 in Western blot assay (46). In the studies mentioned above, observation of bands of higher molecular weight than expected could be due to factors such as protein aggregation, incomplete denaturation or residual disulfide bonding. In fact, GAP is suggested to form a helix-loop-helix structure (47), which may affect Western blotting results. Hence, our results of Western blot assay are like those reported in other fish species. Further, our pre-absorption study using respective GAP antigens successfully abolished the GAP immunoreactivities in the brain. Our current and previous single cell RT-PCR showed expression of *gnrh1* in GnRH-GAP1 cells, *gnrh2* in GnRH-GAP2 cells and *gnrh3* in GnRH-GAP3 cells (38, 39), confirming genetic characteristics of GAP-ir cells as GnRH cells. In addition, the localization patterns of GAP-ir cells completely overlapped with cells expressing respective GnRH mRNA in the *in situ* hybridization study and GnRH peptide synthesizing cells in the immunocytochemical study (34, 41). Although there remains a low possibility of cross-reaction of the three GAP antisera to some unknown proteins with high molecular weight, but our present and past studies strongly suggest that the immunoreactivity to the three GAP types is highly specific.

The three GAP antisera used in this study specifically immunoreacted with their respective GAP types without cross-reactivity in the brain. Double-immunofluorescence labeling showed the proximities of GAP1- and GAP3-ir neuronal processes and cell somata, which suggests possible functional interaction between GnRH-GAP1 and GnRH-GAP3 neurons. In fact, the density of GnRH-GAP1-ir and GnRH-GAP3-ir neuronal processes, closely located to GnRH-GAP1-ir and GnRH-GAP3-ir cell somata was higher in mature males as compared to immature males.

At the single cell level, all three GnRH-GAP cell types expressed multiple GnRHR gene types, in particular, *gnrhr1* and *gnrhr2*, which is in good agreement with our previous immunohistochemical localization of GnRHR types in GnRH3 cells in the tilapia (48) and mRNA expression of two GnRHR types in GnRH3 cells in the dwarf gourami (49). These results suggest the roles of GnRHR in autocrine/paracrine regulation of GnRH cell types as has demonstrated by electrophysiological studies (50). Although the ligand-receptor interaction of three GnRH and GnRHR types in the tilapia still remains unclear, in medaka, which possesses three GnRHR types that are structurally similar to three tilapia GnRHR types, GnRHR1 and GnRHR2 share similar potencies to GnRH ligands with GnRH2 > GnRH3 > GnRH1 (51). Based on the affinity of medaka GnRHR types against three GnRH types, it can be speculated that GnRH1 and GnRH3 cells could be sensitive to multiple GnRH types through GnRHR1 and GnRHR2 signaling. Although GnRH2 is the most potent ligand for all three GnRHR types in fish (52), there was no proximities of GnRH-GAP1 or GnRH-GAP3 neuronal processes with GnRH-GAP2 cell soma, except GnRH-GAP2 neuronal processes were closely located to GnRH-GAP3 cell somata. This suggests that functional interaction between GnRH1 or GnRH3 with GnRH2 could be limited. Alternatively, GnRH2 could reach its target GnRH cell types via non-synaptic and disparate delivery mechanism such as through the circulation as has been demonstrated in the pituitary of tilapia (53, 54).

In GnRH-GAP3 cells, *gnrhr1* and *gnrhr2* mRNA levels were higher in immature as compared to mature males. In contrast, in GnRH-GAP1 and GnRH-GAP2 cells, *gnrhr2* mRNA levels were significantly higher in mature males, while GnRH-GAP1 neuronal processes with GnRH-GAP3 cell somata were more abundant in mature males as compared to immature males. These observations suggest that GnRHR1 and GnRHR2 could regulate different GnRH cell types under different reproductive conditions. In mature males, GnRHR types could be involved in para or endocrine control by different GnRH peptides, while in immature males, GnRHR types may be participating in autocrine control. In some fish species, GnRH neural activities and physiology are closely associated with social hierarchy. In male *Astatotilapia burtoni*, the size of GnRH1 cell soma changes reversibly depending on male social status (55). In medaka fish, the expressions of *gnrh1* is higher in dominant than in subordinate males, while *gnrh3* expression is higher in subordinate than in dominant males (56). In the tilapia, immuno-suppression of GnRH3 results in reduction of male social behaviors (27). However, social hierarchy in male cichlid fish is more for territory defense (aggressiveness), access to food resources (body mass) and securing future mating opportunity (57–60). Therefore, it is likely that GnRH-GAP1 is mainly responsible for the reproduction (61, 62), GnRH-GAP2 is for food intake/metabolism (22, 23), and GnRH-GAP3 is for socio-sexual behaviors (26–28), but they might coordinate with each other. Nevertheless, in the future, it will be interesting to study the three GnRH-GAP changes in one species at the same time.

Although the autocrine regulation of GnRH neurons has been considered a mechanism for the pulsatile secretion of GnRH (31), the physiological significance of expression of multiple GnRHR types in GnRH neurons as well as paracrine or autocrine regulation of GnRH neurons in other functions remains unclear. Some studies have implied a possible role of autocrine regulation of GnRH. For example, during developmental stages of zebrafish, GnRH3 peptides have been shown to regulate development of GnRH3 neurons in an autocrine fashion (63). In hypothalamic GT1-1 neuronal cells, GnRH has been shown to exert autocrine regulation at the level of GnRH gene transcription (64). Interestingly, in the zebrafish embryo, gene knockdown of *gnrh3* affected regionalization of the brain and eye formation (65). Therefore, these non-reproductive functions of GnRH3 could also be regulated by multiple GnRH types in an autocrine and/or paracrine manner during sexual maturation.

The present study shows morphological and molecular evidences for the possibility of homogenous interaction within the GnRH-GAP neural group as well as heterogeneous interaction among different GnRH-GAP cell types. Co-expression of multiple GnRHR types in GnRH-GAP cell types may also suggest the possibility of heterodimerization of GnRHR types. In the protochordate, *Ciona intestinalis*, GnRHR heterodimer has been found to regulate various physiology of GnRH cells such as the elevation of intracellular calcium, time-extension of ERK phosphorylation, and up-regulation of cell proliferation (66). GPCR dimerization are also known to promote receptor folding, maturation and/or transport to the cell surface (67). Therefore, the presence of multiple GnRHR types and their functional interaction could also be important for not only the regulation of GnRH neuronal activity, but also the regulation of GnRHR cell-surface expression, which might influence on the probability of ligand-receptor binding under different physiological conditions. Autocrine/paracrine mechanisms have also been suggested to play an important role as a pulse generator (31). However, there is no direct evidence of coordinated release of GnRH by the different GnRH-GAP types in the non-mammalian species. We have previously demonstrated that inhibition of GnRH3 system influences the outcome of GnRH1 and GnRH2 in a cichlid (27). The present study provides a morphological evidence for the potential interrelationship between the hypophysiotropic and non-hypophysiotropic GnRH systems, which might be involved in co-modulation of reproductive and non-reproductive events.

## Data Availability Statement

The datasets generated for this study are available on request to the corresponding author.

## Ethics Statement

The animal study was reviewed and approved by Animal Ethics Committee of Nippon Medical School.

## Author Contributions

SO and IP: conceptualization, data analysis, and funding acquisition. SO: investigation and writing—original draft preparation. IP: writing—review and editing. All authors contributed to the article and approved the submitted version.

## Conflict of Interest

The authors declare that the research was conducted in the absence of any commercial or financial relationships that could be construed as a potential conflict of interest.
