# Supplementary material for: Morphological Evidence for Functional Crosstalk Between Multiple GnRH Systems in the Male Tilapia, Oreochromis niloticus
Source: Front Endocrinol (Lausanne). 2020 Sep 2;11:586. doi: 10.3389/fendo.2020.00586 (PMC7492274; doi:10.3389/fendo.2020.00586)
Supplement: Supplementary file 1 [file Table_1.docx]

**Supplementary Table 1**

Primers and fluorogenic probes used for real-time PCR for GnRH and GnRH receptor subtypes genes.

| **Gene** | **Forward (5’-3’)** | ***Probe (5’-3’)** | **Reverse (5’-3’)** | **GenBank Accession No** |
| --- | --- | --- | --- | --- |
| *gnrh1* | CTCGCAGGGACGGTGTTT | CACAGGGCTGCTGTCAACACTGGTCATA | CGGCCAAACTCGCAAGAA | AB101665 |
| *gnrh2* | TGGTCCCATGGTTGGTATCC | AAATCTCTGATGTCCCAAAGGAGTCCAGCT | GGCTAAGGCATCCAGAAGAATGT | AB101666 |
| *gnrh3* | TGCTGGCGTTGGTGGTT | CAGCACTGGTCCTATGGATGGCTACC | CCTGTGCCCATCATCCTAATG | AB101667 |
| *gnrhr1* | TCCAGACGACTGAAAAAGGACA | TTTACCTTCCAGTGAAATGCACCTGCG | GAGTTCTCATCCGGGCTCTC | AB111356 |
| *gnrhr2* | ATCCACCAGCAGCACCTGA | GAGACAAAGCAGGTGAGTCGCCCCTG | ATGTCGGTGCCACTGCG | AB111357 |
| *gnrhr3* | TGCTACACCAGGATCTTTATTCAGA | ACAGATGACAAAAAAGAACATGCCCTCCAAT | GAGCAGCGGAGATGTGGC | AB158490 |
| *gfap* | GGCACTGAAAGAGGAGATGG | GCAGGATCACGATTCCAGTT | CATGGGCCTCAGGAGTTTTA | XM_003441987 |

*All probes were labeled with 5’ FAM for reporter dye and 3’ TAMARA for quencher dye.
